# Commentary: Case report: Optic atrophy and nephropathy with m.13513G>A/MT-ND5 mtDNA pathogenic variant

**DOI:** 10.3389/fgene.2022.988122

**Published:** 2022-11-28

**Authors:** Josef Finsterer

**Affiliations:** Neurology & Neurophysiology Center, Vienna, Austria

**Keywords:** mtDNA, complex-I, LHON, multisystem, heteroplasmy, m.13513G>A

Letter to the editor

We read with interest the article by Barone et al. about a 48 years old male with a multisystem mitochondrial disorder (MID) due to the variant m.13513G>A in *ND5* (Barone et al., 2022). Phenotypically, the patient manifested with subcortical cerebellar atrophy, mineralisation of the globus pallidus, Leber’s hereditary optic neuropathy (LHON), optic atrophy, hearing loss, and nephropathy (Barone et al., 2022). The study is appealing but raises concerns that should be discussed.

Many MIDs manifest phenotypically with cardiac disease (Lioncino et al., 2022). This is also the case with patients carrying the m.13513G>A variant (Ruiter et al., 2007). Cardiac manifestations in m.13513G>A carriers include cardiomyopathy and Wolff-Parkinson-White (WPW) syndrome. Therefore, it would be highly valuable to know the results of the clinical cardiologic examination, ECG, transthoracic echocardiography, the cardiac stress test, and eventually cardiac MRI.

Like many other MIDs, conditions due to the variant m.13513G>A may also manifest in the skeletal muscles as myopathy, ptosis, or ophthalmoparesis (Wei et al., 2021). Therefore, it would be interesting to know the results of the clinical neurologic examination, serum creatine-kinase, lactate-dehydrogenase, aldolase, myoglobin and lactate levels, and needle electromyography studies, and eventually muscle biopsy. This will definitely enhance the quality and clarity of case presentation.

According to a recent review of 50 patients carrying the m.13513G>A variant, the spectrum of the m.13513G>A variant is much broader than anticipated, and also includes fatal neonatal acidosis, renal dysplasia, cardiomyopathy, WPW syndrome, strabism, progressive external ophthalmoplegia, seizures, stroke-like episodes (SLEs), ataxia, tremor, and psychosis (Yahata et al., 2017) (Table 1). Therefore, it would be highly valuable to know if electroencephalography was normal and if any of these features were present in the index patient.

**TABLE 1:** Phenotypic manifestations of the variant m.13513G>A in the index patient and the literature so far reported.

Phenotypic feature	Index patient	Literature	Reference
Nephropathy	Yes	No	[none]
Calcified globus pallidus	yes	No	[none]
Cerebellar atrophy	Yes	yes	[Wei]
LHON	Yes	yes	[Vázquez-Justes]
Hearing loss	Yes	yes	[Shanske]
Optic atrophy	Yes	yes	[Kirby]
Cardiomyopathy	No	yes	[Vázquez-Justes]
Myopathy	No	yes	[Vázquez-Justes]
Strabism	No	yes	[Ruiter]
WPW syndrome	No	yes	[Liang]
Nystagmus	No	yes	[Ruiter]
Seizures	No	Yes	[Sudo]
Ataxia	No	Yes	[Sudo]
Fatal neonatal acidosis	No	Yes	[van Karnebeck]
Renal dysplasia	No	Yes	[van Karnebeck]
Tremor	No	Yes	[Sudo]
Exotropia	No	Yes	[Kirby]
PEO	No	Yes	[Kirby]
MELAS	No	yes	[Shankse]
Leigh syndrome	No	yes	[Shankse]

LHON, Leber’s hereditary optic neuropathy; PEO, progressive external ophthalmoplegia; WPW-syndrome, Wolff-Parkinson-White syndrome.

Though the combination of renal insufficiency, cerebral atrophy, LHON, and hypoacusis, may have been undescribed prior to the index case, the single individual features are all well-known from m.13513G>A carriers (Table 1). LHON is a common feature of m.13513G>A carriers, as well as hypoacusis, and cerebral atrophy. Only renal failure has been found only in a few patients with the m.13513G>A variant (Yahata et al., 2017).

We disagree with the consideration that the reduced MMSE could be due to visual impairment (Barone et al., 2022). Because m.13513G>A carriers frequently manifest with cerebral atrophy, like the index patient, and with psychiatric disease, the MMSE score of 28 is more likely attributable to cerebral than ophthalmologic involvement. Furthermore, MR-spectroscopy showed cerebral lactic acidosis, suggesting that cognitive impairment may also derive from cerebral acidosis.

Bilateral T2-hyperintensities in the globus pallidus, which were hypointense on susceptibility weighted imaging (SWI) were interpreted as mineralisation (Barone et al., 2022). It would be highly valuable to know whether the cerebral CT scan showed basal ganglia calcifications or if these lesions were due to increased iron deposition.

Overall, the interesting study has some limitations that call the results and their interpretation into question. Clarifying these weaknesses would strengthen the conclusions and could improve the study. The phenotypic spectrum of m.13513G>A carriers is broader than anticipated.
